# HLA-B8 association with late-stage melanoma – an immunological lesson?

**DOI:** 10.1186/1741-7015-4-5

**Published:** 2006-03-13

**Authors:** Joachim Fensterle, Uwe Trefzer, Thomas Berger, Mads Hald Andersen, Selma Ugurel, Jürgen C Becker

**Affiliations:** 1 Inst. f. Med. Strahlenkunde und Zellforschung MSZ, University Clinics of Würzburg, Versbacher Str. 5, 97078 Würzburg, Germany; 2 Department of Dermatology, Charité Universitätsmedizin, Berlin, Germany; 3 Department of Dermatology, University Clinics of Erlangen, Germany; 4 Danish Cancer Society, Copenhagen, Denmark; 5 Department of Dermatology, University Clinics of Mannheim, Germany; 6 Department of Dermatology, University Clinics of Würzburg, Würzburg, Germany; 7 Zentaris GmbH, Frankfurt, Germany

## Abstract

**Background:**

Differences in HLA allele frequencies between the diseased and healthy populations may signify efficient immune responses, a notion that has been successfully tested for infectious diseases or for association with genetic elements involved in a distinct type of immunity. This retrospective study is intended to detect differences in MHC class I carrier frequencies of advanced melanoma patients compared to healthy bone marrow donors.

**Methods:**

The HLA-A and -B carrier frequencies of 748 stage IV melanoma patients retrieved from serotyping at 6 different centers in Germany were compared using a chi-square test to 13,386 fully HLA typed bone marrow donors registered in the German national bone marrow donor registry.

**Results:**

The comparison of HLA carrier frequencies in advanced cancer patients with healthy bone marrow donors revealed a significant decrease in HLA-B8 carrier frequencies, which was also apparent in patients with advanced disease compared to patients with loco-regional disease.

**Conclusion:**

The data suggest that protective immune responses restricted to distinct MHC class I molecules may be operational in a subset of melanoma patients, which is the prerequisite for a large scale screen for the corresponding epitopes. Alternatively, the known association of the ancestral haplotype HLA-A1, -B8 and -DR3 with genetic elements such as distinct TNF-α alleles might have a protective effect on disease progression. In any case, identification of the cause of protection within this patient subset might lead to a significant improvement in the efficacy of current immunotherapeutic approaches.

## Background

Active immunization against solid tumors is amongst the most promising therapeutic approaches for such diseases. Different immunization schedules are currently being explored in numerous clinical trials, including vaccination with MHC class I restricted T-cell epitopes derived from tumor associated antigens (TAA) [1]. To date, oncogenes have been intensively studied as targets for immunotherapy to minimize the risk of immune escape. As an example, we have recently identified B-Raf V600E (previously designated B-Raf V599E) as a potential TAA for immunotherapies against melanoma [2,3]. The tumor antigens deployed are characterized by their ability to elicit immune responses in patients, and the choice of peptide epitopes derived from these antigens is mainly dictated by the frequency of HLA alleles in the normal population [4]. However, this strategy for identifying targets for therapy might actually miss the most valuable epitopes.

As early as 1991, it was proposed that disease-associated imbalances in HLA allele frequencies between diseased and healthy populations may signify efficient immune responses [5]. Although this approach was successfully adapted for the malaria system [6], transfer to cancer research has not yet been envisioned. In theory, any epitope derived from TAA that elicits an effective immune response able to eradicate the tumor should impact on the frequency of the respective HLA allele in this patient population. Assuming that the antigen is reasonably common within the patient cohort, the frequency of the HLA allele in the context of which the epitope is presented should be substantially reduced in patients with progressive disease, i.e. those who failed to mount an effective immune response. In practice, it is difficult to validate this edifice of ideas, as several prerequisites usually remain unknown: (i) the nature and (ii) the grade of expression of the corresponding TAA, and (iii) the percentage of patients mounting a protective immune response against this antigen. The fact that the HLA phenotype is known for a large number of patients suffering from advanced stages of immunogenic tumors such as melanoma allows at least one consequence of this intellectual experiment to be verified or falsified: viz., whether the frequency of certain HLA class I alleles is reduced in patients in whom the immune system failed to control the disease.

However, HLA imbalances can also be explained by linkage to genetic factors influencing disease progression. In this respect, it should be noted that many non-MHC genes encoding immunologically relevant molecules such as TNF-α or components of the complement system are located in the HLA locus, and that some alleles of these non-MHC genes are genetically linked to certain HLA alleles.

## Methods

### Data acquisition and HLA serotyping

The results of the serological HLA-A and -B typing of 748 AJCC stage IV melanoma patients performed in the University Hospitals of Erlangen (267 patients), Berlin (217 patients), Würzburg (141 patients), Mannheim (82 patients), Mainz (26 patients) and Münster (15 patients) were collected. To compare stage I-III with stage IV patients, the serotyping of 302 stage I-III patients performed in Berlin was also taken into account. The scientific workup of the HLA phenotyping results was approved by the local ethics committees, provided the patient gave informed consent.

### Data comparison and statistical analysis

The data compiled from this patient cohort were compared to those from 13,386 fully HLA-typed bone marrow donors registered in the German national bone marrow donor registry [7]. Data analysis was performed using the Graph Pad Prism software package and MS Excel 2003. Stage IV patients were compared to healthy bone marrow donors using a two sided chi-square test; stage I-III patients were compared to stage IV patients from Berlin, and to the whole cohort, using a two sided Fisher's exact test. An observation was regarded as significant if p < 0.01. The ratio between stage IV and stage I-III carrier frequencies was calculated to compare the relative decrease between different HLA alleles; the mean ratio comprises all HLA alleles with carrier frequencies above 15% (n = 11) +/- standard deviation.

## Results and discussion

Table 1 summarizes the HLA frequencies for MHC class I alleles of the HLA-A, HLA-B and HLA-C loci in stage IV melanoma patients. Frequencies are given for all alleles that have been typed. If subtypes (depicted in italics) were determined, the main type (depicted in bold) contains the cumulative carrier frequencies and the percentage of subtypes is given. Because HLA-C was only phenotyped in approximately half the patients, only the HLA-A and -B carrier frequencies were compared to healthy blood donors, and we restricted our analysis to HLA alleles with carrier frequencies above 4.5% (table 2). Subtypes were compared if more than 90% of the corresponding allele were subtyped in both groups. While no significant variation between the two groups was evident for most alleles, the frequency of HLA-B8 exhibited a significant decrease from 20.3% in bone marrow donors to 16.2% in melanoma patients (P = 0.0079, two-sided chi square test). In addition, HLA-B35 and HLA-A26 showed a non-significant tendency towards lower carrier frequencies (HLA-B35 from 18.5% to 15.2%; P = 0.027, and HLA-A26 from 6.98% to 5.01%; P = 0.039). The statistical analysis was performed using a two sided chi-square test and P values below 0.01 were regarded as statistically significant. The lower P value was chosen to avoid type I errors due to multiallele comparison, according to the suggestion by Svejgaard and Ryder [8]. In contrast, a Bonferroni correction for an hypothesis-generating approach in a retrospective study like this, with no a priori hypothesis for a specific association, is not indicated [9].

**Figure 1:**
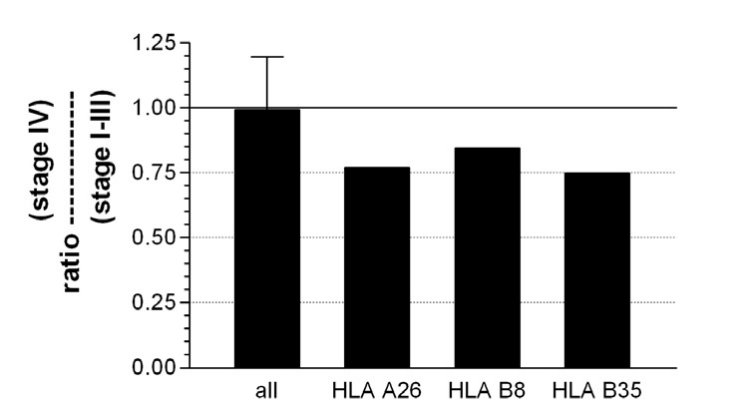
**Decrease of HLA-A26, HLA-B8 and HLA-B35 carrier frequencies in advanced melanoma patients**. The ratios between HLA carrier frequencies of stage IV patients and stage I-III patients are calculated to compare relative changes in carrier frequencies from data acquired in one center; all: mean ratio of all HLA-A and -B alleles with carrier frequencies >15%.

To analyze sufficient numbers of stage IV melanoma patients, we pooled data sets from different dermatology departments throughout Germany. Although this approach has the advantage of minimizing the likelihood of systematic errors from the data acquisition process, it would be still of interest to compare data sets derived from the same center. This would also help to overcome the necessity to compare stage IV melanoma patients with healthy bone marrow donors, with no further information on parameters such as skin type or ethnic background, by using the more appropriate control of early disease patients. Consequently, in one center, additional HLA data were acquired from stage I-III melanoma patients. Thus, it was possible to compare HLA-A and -B carrier frequencies of patients with loco-regional disease (n = 302 HLA-A, n = 301 HLA-B) with those of disseminated melanoma (n = 214 HLA-A, n = 215 HLA-B). As depicted in Fig. 1, stage IV patients are again characterized by a decrease in carrier frequencies for HLA-A26, HLA-B8 and HLA-B35, whereas the means of the other HLA alleles remain stable. The difference is in the same range as that observed when the initial cohort of stage IV patients was compared with healthy bone marrow donors. It should be noted, however, that the observed reduction was not statistically significant for either HLA-B8 (stage I-III: 20.93%, stage IV: 17.67%, P = 0.37 two sided Fisher's exact test), HLA-B35 (stage I-III: 19.27%, stage IV: 14.42%, P = 0.16) or HLA-A26 (stage I-III: 7.28%, stage IV: 5.61%, P = 0.48), mainly because of the small number of patients included in this analysis, leading to a low statistical power. However, differences in ethnic background within the melanoma patient and bone marrow donor groups might still be a concern. Indeed, the 8.1 ancestral haplotype (8.1 AH) encompassing the alleles HLA-A1, -B8 and DR3 is much more frequent in the Caucasian population than in other ethnic groups. As an example, Cao et al. compared the HLA frequencies of the five major ethnic groups in the US population [10]. In their study, HLA-B8 allele frequencies were estimated as 10.9% for Caucasians, with values ranging from 1.2% (Asians) to 4.6% (African Americans) for the other ethnic groups. Although these values cannot be translated into the skin types of German bone marrow donors, it is obvious that all ethnic groups having a darker skin type have significantly lower HLA-B8 allele frequencies than Caucasians. Therefore, an under-representation of these ethnic groups in stage IV melanoma patients should lead to an increased, rather than a reduced, HLA-B8 frequency when compared to the normal population. Taken together, these arguments do not support the notion that differences in skin type between the melanoma patient and control groups account for the observed reduction in HLA-B8 carrier frequencies.

It should be noted that several independent reports have already demonstrated a reduction of HLA-B8 and HLA-B35 in melanoma patients [11-13] or a selective loss of HLA alleles in metastasis [14]. Furthermore, HLA-B8- and HLA-B35-restricted melanoma-specific T cell responses have been identified [15,16]. The recognized epitopes are derived from tyrosinase, Melan-A/MART-1, gp100, MAGE-A3/MAGE-A6 and NY-ESO-1 [17-19]; we recently added a HLA-B35-restricted epitope of the inhibitor of apoptosis protein survivin to this list [20]. Thus, it is tempting to speculate that the observed decrease of HLA-B8 carrier frequencies in advanced melanoma is the result of an effective immune response directed against specific TAA-derived epitopes presented by the respective HLA restriction elements, or, conversely, the lack of such response in patients not expressing these restriction elements. However, whether the epitopes already identified are indeed the targets for the hypothetical, protective immune response responsible for the observed decrease in HLA frequencies remains elusive. Such epitopes could only be defined by quantitative and qualitative comparison of the cellular immune responses between patients with early disease, preferentially the primary tumor, and those with advanced disease; in the latter patients, the protective responses should be underrepresented.

An alternative explanation for the observed decrease in HLA frequencies could be the genetic linkage to a tumor suppressor in the broadest sense. HLA-B8 has been shown to be linked genetically to HLA-A1 [21]. This linkage is also obvious in our data set of stage IV melanoma patients, where 80.8% of the patients carrying HLA-B8 are also positive for HLA-A1. Although no gene dosage effect that could translate into a reduced number of homozygous HLA-B8 carriers in the patient group was observed (data not shown), the association of the 8.1 ancestral haplotype with autoimmune diseases or distinct TNF-α or complement alleles might be of special interest, as this might exert an influence on the anti-tumoral immune response [22-24]. In the case of TNF-α, the -308A allele that is linked with 8.1 AH is associated with altered TNF-α production *in vitro *[22]. Also, 8.1 AH is characterized by a non-functional C4B allele and the lack of the C4A gene, translating into defective complement function [25,26]. As the complement cascade is involved in inflammation, the clearance of immune complexes, antibody-dependent cellular cytotoxicty and other immunologically relevant processes, a genetic defect in the complement system may indeed affect both innate and adaptive immunity. However, the definitive answer to whether HLA-B8 itself or genetic elements linked to HLA-B8 are responsible for the observed protective phenotype can only be elucidated by further studies, including the study of TNF-α or complement alleles.

## Conclusion

Overall, this study suggests a significant decrease of HLA-B8 carrier frequencies in melanoma patients compared to healthy donors. This difference, which was observed when HLA carrier frequencies in advanced cancer patients were compared with healthy bone marrow donors or patients with loco-regional disease, supports the hypothesis that protective immune responses may be operational in a subset of melanoma patients independently of therapy, and that distinct MHC class I restriction elements seem to be preferentially involved in such responses. Alternatively, the association of 8.1 AH with genetic elements such as distinct TNF-α alleles or defective complement components might result in an altered quality of immune response, preventing the progression of the disease. If the observed differences can be verified in a follow-up study, a search for the corresponding T-cell epitopes and/or the associated genetic elements is indicated and might lead to a new generation of effective tumor vaccines.

## Competing interests

Since May 2005, JF has been an employee of Zentaris GmbH, Frankfurt, Germany.

## Authors' contributions

JF and JCB designed the study and discussed the results of the statistical analysis. JF performed the statistical analysis and drafted the manuscript. JCB coordinated data acquisition across the different centers. UT, TB, SU, MHA and JCB supervised and coordinated data collection in the corresponding dermatology departments. All authors approved the final version of the manuscript.

## Pre-publication history

The pre-publication history for this paper can be accessed here:



## Figures and Tables

**Table 1:** HLA-A, -B and -C carrier frequencies of stage IV melanoma patients. HLA-A and B alleles of 739 and 709 stage IV melanoma patients were typed serologically; HLA-C (391 stage IV melanoma patients) was typed by PCR. The frequencies of all alleles included in the analysis are shown. Subtypes are given in italics; the main types that have been partially subtyped are shown in bold. HLA carrier frequencies of the main types that have been partially subtyped encompass the carrier frequencies of the subtypes; the fraction of patients who have been subtyped for a certain allele is given as a percentage.

HLA-A (n = 739)			HLA-B (n = 709)			HLA-C (n = 391)	
serotype	frequency	subtyped	serotype	frequency	subtyped	allele	frequency
HLA-A1	28.69%		**HLA-B5**	12.55%	89.89%	HLA-Cw1	5.88%
HLA-A2	51.42%		*HLA-B51*	10.30%		HLA-Cw2	12.53%
HLA-A3	28.96%		*HLA-B52*	0.99%		HLA-Cw3	23.02%
**HLA-A9**	23.68%	99.43%	HLA-B7	27.79%		HLA-Cw4	23.53%
*HLA-A23*	5.41%		HLA-B8	16.22%		HLA-Cw5	12.79%
*HLA-A24*	18.13%		**HLA-B12**	24.96%	98.31%	HLA-Cw6	23.27%
**HLA-A10**	11.23%	98.80%	*HLA-B44*	23.41%		HLA-Cw7	48.34%
*HLA-A25*	5.55%		*HLA-B45*	1.13%		HLA-Cw8	3.07%
*HLA-A26*	5.01%		HLA-B13	5.64%			
*HLA-A34*	0.14%		**HLA-B14**	4.80%	29.41%		
*HLA-A66*	0.41%		*HLA-B64*	0.28%			
HLA-A11	9.34%		*HLA-B65*	1.13%			
**HLA-A19**	22.46%	96.99%	**HLA-B15**	14.10%	79.00%		
*HLA-A29*	5.82%		*HLA-B62*	10.58%			
*HLA-A30*	2.98%		*HLA-B63*	0.56%			
*HLA-A31*	5.14%		*HLA-B75*	0.00%			
*HLA-A32*	5.55%		**HLA-B16**	9.59%	98.53%		
*HLA-A33*	2.30%		*HLA-B38*	4.94%			
**HLA-A28**	6.63%	36.73%	*HLA-B39*	4.51%			
*HLA-A68*	2.03%		HLA-B18	10.58%			
*HLA-A69*	0.41%		**HLA-B21**	4.65%	96.97%		
			*HLA-B49*	2.26%			
			*HLA-B50*	2.26%			
			**HLA-B22**	3.24%	95.65%		
			*HLA-B54*	0.14%			
			*HLA-B55*	1.41%			
			*HLA-B56*	1.55%			
			HLA-B27	8.89%			
			HLA-B35	15.23%			
			HLA-B37	3.95%			
			**HLA-B40**	11.99%	90.59%		
			*HLA-B60*	8.46%			
			*HLA-B61*	2.40%			
			HLA-B41	2.96%			

**Table 2:** Comparison of HLA-A and B carrier frequencies between stage IV melanoma patients and healthy bone marrow donors. HLA-A and -B carrier frequencies of stage IV melanoma patients (739 HLA-A; 709 HLA-B) and healthy bone marrow donors (13,386) were compared using a two sided chi-square test with a 95% confidence interval and sorted according to the absolute difference. Results are regarded as significant for p values < 0.01; alleles with significant changes are depicted in bold; results are sorted according to the percentage difference. Only alleles with carrier frequencies > 5% are included. Subtypes are only included if more than 89% of the corresponding allele was subtyped in the melanoma and bone marrow donor group. OR: odds ratio; ci: confidence interval.

HLA	melanoma	control	difference	p	sign.	OR	95% ci OR
**B8**	**16.22%**	**20.33%**	**4.12%**	**0.0079**	*	**0.76**	**0.6184 to 0.9302**
B35	15.23%	18.53%	3.29%	0.0273	ns	0.79	0.6409 to 0.9744
B7	27.79%	25.34%	2.45%	0.1452	ns	1.13	0.9575 to 1.342
A9	23.68%	21.42%	2.26%	0.1452	ns	1.14	0.9561 to 1.356
A10	11.23%	13.33%	2.1%	0.1015	ns	0.82	0.6509 to 1.039
A26	5.01%	6.98%	1.97%	0.0393	ns	0.70	0.5015 to 0.9846
B40	11.99%	13.84%	1.85%	0.1624	ns	0.85	0.6728 to 1.070
B60	8.46%	10.32%	1.85%	0.1123	ns	0.80	0.6133 to 1.053
B15	14.1%	15.8%	1.7%	0.2266	ns	0.88	0.7047 to 1.087
A28	6.63%	8.3%	1.67%	0.1076	ns	0.78	0.5834 to 1.055
B16	9.59%	7.94%	1.65%	0.1151	ns	1.23	0.9504 to 1.591
B13	5.64%	6.96%	1.31%	0.1762	ns	0.80	0.5774 to 1.108
A32	5.55%	6.69%	1.14%	0.2232	ns	0.82	0.5939 to 1.132
B12	24.96%	23.83%	1.13%	0.4902	ns	1.06	0.8930 to 1.266
A11	9.34%	10.46%	1.12%	0.3308	ns	0.88	0.6839 to 1.137
A24	18.13%	17.26%	0.88%	0.5401	ns	1.06	0.8761 to 1.287
A3	28.96%	28.1%	0.85%	0.6122	ns	1.04	0.8856 to 1.228
B18	10.58%	9.79%	0.79%	0.4900	ns	1.09	0.8526 to 1.395
A19	22.46%	21.79%	0.67%	0.6671	ns	1.04	0.8705 to 1.242
B44	23.41%	22.78%	0.63%	0.6942	ns	1.04	0.8666 to 1.238
A2	51.42%	51.13%	0.29%	0.8768	ns	1.01	0.8724 to 1.173
A1	28.69%	28.94%	0.25%	0.8825	ns	0.99	0.8385 to 1.163
B51	10.3%	10.54%	0.24%	0.8361	ns	0.97	0.7598 to 1.249
B27	8.89%	8.73%	0.15%	0.8884	ns	1.02	0.7814 to 1.329
A25	5.55%	5.61%	0.06%	0.9429	ns	0.99	0.7151 to 1.366
